# Long-Segment Versus Short-Segment Pedicle Screw Fixation Including Fractured Vertebrae for the Management of Unstable Thoracolumbar Burst Fractures

**DOI:** 10.7759/cureus.35235

**Published:** 2023-02-20

**Authors:** Abdullah Al Mamun Choudhury, Md. Shah Alam, Sharif Jonayed, OZM Dastagir, Md. Sarwar Jahan

**Affiliations:** 1 Spine Surgery, National Institute of Traumatology & Orthopaedic Rehabilitation, Dhaka, BGD; 2 Spine Surgery, Bangladesh Spine & Orthopaedics Hospital, Dhaka, BGD; 3 Orthopaedics and Traumatology, National Institute of Traumatology & Orthopaedic Rehabilitation, Dhaka, BGD

**Keywords:** motion-preserving spine surgery, comparative study, incomplete sci, index level fixation, thoracolumbar fracture

## Abstract

Background

For the treatment of unstable thoracolumbar fractures, this study compared the results of short-segment fixation with fracture level inclusion (SSFIFL) with long-segment pedicle fixation (LSPF).

Methodology

In this prospective case series study conducted from January 2015 to January 2019, 80 patients with partial spinal cord lesions were investigated. For the comparison, two groups of 40 patients each were chosen and treated with SSFIFL and LSPF. The outcomes were measured using pre and postoperative radiological parameters and clinical parameters. The radiographic variables included the kyphotic angle with loss of correction, kyphotic deformation, and the Beck index. Mean blood loss, operative time, and cost-effectiveness were also examined for clinical indicators such as the American Spinal Injury Association Impairment Scale, Visual Analog Scale (VAS), and Oswestry Disability Index (ODI).

Results

There were no substantial variations between the groups regarding age or gender, trauma etiology, fracture level, or fracture pattern. Between the two categories, there appeared to be no notable change in radiological indicators such as kyphotic angle, kyphotic deformation, and Beck index at the end of follow-up (p = 0.120, 0.360, and 0.776, respectively). Both groups had similar neurological outcomes (p = 0.781). In terms of ODI and VAS, statistically, there was no discernible difference (p = 0.567 and 0.161, respectively). In this study, however, there was less surgical time, blood loss, and implant cost (p = 0.05).

Conclusions

When fracture level is included in a short-segment fixation, the radiological and clinical results are comparable to long-segment posterior fixation. Ultimately, this treatment has proven to be not only a motion segment-saving procedure but also cost-effective.

## Introduction

The debate on the treatment of thoracolumbar fractures has been ongoing for a long time [1-3]. Despite this, the majority of authors think that surgery should be performed for unstable burst fractures. However, the methods remain debatable [3-5].

There is no difference in efficacy between the anterior and posterior techniques, regardless of which technique is chosen [1,6]. However, the posterior approach is considered to be less hazardous [7,8] and preferable in stabilizing acute unstable thoracolumbar fractures [9].

Surgery aims to obtain the most stable fixation as well as a neural canal decompression [1,4,5,10,11]. However, to preserve the maximal thoracolumbar motion, the number of fusion segments needs to be reduced [12,13].

Recently, short-segment stabilization with pedicle screws (SSPF) involving one level down and one level up from the fractured vertebra has become popular because of its easier application, fewer implant use, reduced blood loss, and smaller incision [3,4,14-21]. Incorporating fewer motion segments than long-segment pedicle fixation (LSPF) is the main advantage [4,21], but in the long run, SSPF can result in implant failure [8] and re- kyphosis (9-54%) [4,5].

Insufficient anterior column support, inadequate fixation due to osteoporosis, and inadequate level of fixation are the most likely explanations for failures [22]. More level incorporation allows each pedicle screw to be less stressed, which was accomplished by using long-segment constructs that connect two or three levels up and down from the broken vertebra. This technique has a lower failure rate [14,20,23] but comes at the expense of additional instrumentation and additional complications [14,16,23].

To reduce failure rates, additional anterior column stabilization techniques such as strut grafting, mesh cage plates, and transpedicular bone grafting with vertebroplasty or kyphoplasty with calcium phosphate cement or polymethyl methacrylate [14,24] have been introduced.

Incorporating fractured vertebrae to spread the load while restricting the number of motion segments fused [16,23] is another option. In patients with thoracolumbar burst fractures, this strengthening displays superior kyphosis correction and instant stability [16] and theoretically reduces the number of fused motion segments.

Some studies have analyzed LSPF to SSPF or SSPF to anterior column augmentation but none included a fracture screw combination [25,26].

In contrast to LSPF, it was speculated that fracture-level screws coupled with short-segment fixation with fracture level inclusion (SSFIFL) get the same stability. This prospective study aimed to compare the functional and radiological outcomes of short-segment fixation, which included fractured vertebrae, with long-segment pedicle screw fixation for the management of unstable thoracolumbar burst fracture having incomplete neurodeficit.

## Materials and methods

After receiving approval from the Institutional Ethical Board (IRB) Committee (approval number: NITOR/ACADEMY/PHASE-B/5875) of the National Institute of Traumatology & Orthopaedic Rehabilitation (NITOR), this research was conducted in a tertiary hospital. A total of 80 patients aged 15-60 years with unstable thoracolumbar burst fractures and incomplete spinal cord injuries and a load-sharing classification of <6 and clinical instability score ≥5 [27] were included in the study from January 2015 to January 2019. The study excluded patients with head trauma, full neuro-deficiency, polytrauma, pathological fractures, or multilevel injuries.

All patients were given a written explanation of the process, as well as the several therapy options available before they participated in the study. After initial resuscitation by the advanced trauma life support protocol, a detailed history (including sex, age, mechanism of injury, comorbidities, and occupation) was recorded, and a complete examination was done. The patient’s neurological condition was evaluated using the American Spinal Injury Association (ASIA) motor score as well as bladder and bowel function. Subsequently, the patients were randomized into two groups based on a random-allocation sequence, i.e., odd numbers corresponded to the SSFIFL group and even numbers corresponded to the LSPF group. The information needed for this study was gathered by an expert technologist and an analyst who were uninformed of the study’s goals and surgical procedures.

SSFIFL (group I) constructions encompassing two vertebrae to the injury, one upper and one lower, were used to treat 40 patients (32 males and eight females). There were another 40 individuals in group II who were treated with LSPF constructs (35 males and five females). Four vertebrae were treated with instrumentation, two on either side of the fracture, with no screws placed in the fractures.

To repair the kyphotic deformity and drive the injured vertebra ventrally, along with a short-segment transpedicular fixation (SSFIFL) at one level above and one level below the injured segment, an additional lordotic screw was inserted at the fracture level on each side after standard posterior midline approach. The surgery was performed under fluoroscopic control, and, if necessary, the pedicle screw construct was manipulated to address the spinal deformity. If a pedicle was found to be injured in the index vertebra, the corresponding side was omitted for screw insertion. All screws were linked together by two rods bent into a lordotic shape. Before fastening the set screws and using cross-link, a distraction force was exerted depending on the injured endplate. According to the location and diameter of the vertebra, the screws were either 40 or 45 mm long. Screws with a diameter of 5.5 mm were used cephalad to the T11. Younger individuals required smaller screws in diameter and length. Decompression was done by laminectomies in all cases to excise the disc remnants or the retropulsion fragments which remained in the spinal canal or to push them back into the vertebral body. We did not use bone grafts to achieve fusion. Mean blood loss, operative time, and cost of the implant were recorded.

**Figure 1:**
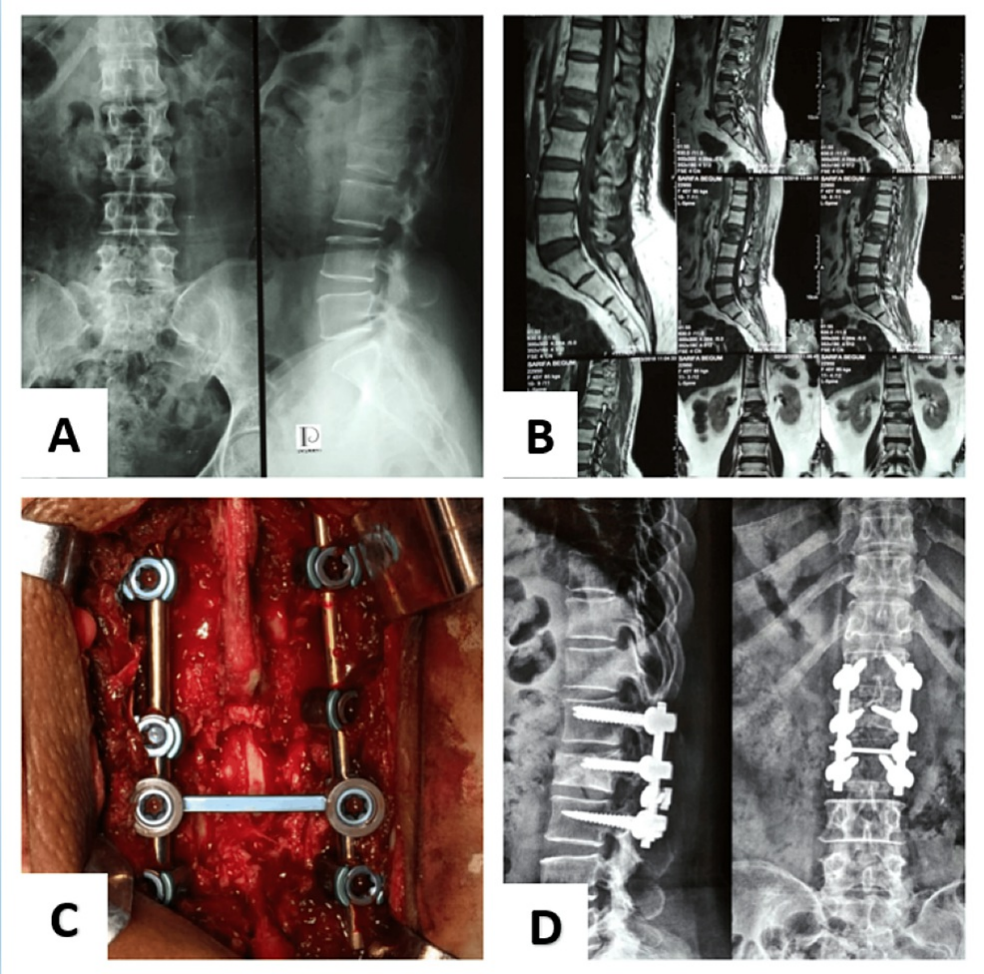
Short-segment fixation including fracture level. A. Preoperative X-ray of the lumbosacral spine anteroposterior and lateral view. B. T2-weighted sagittal section (MRI of the lumbosacral spine) showing burst # L2. C. Peroperative image showing short-segment fixation including fracture level with cross-link. D. X-ray of the lumbosacral spine anteroposterior and lateral view at the final follow-up.

Figure 1 depicts a burst # L2 in a 45-year-old woman that was repaired with SSFIFL.

**Figure 2:**
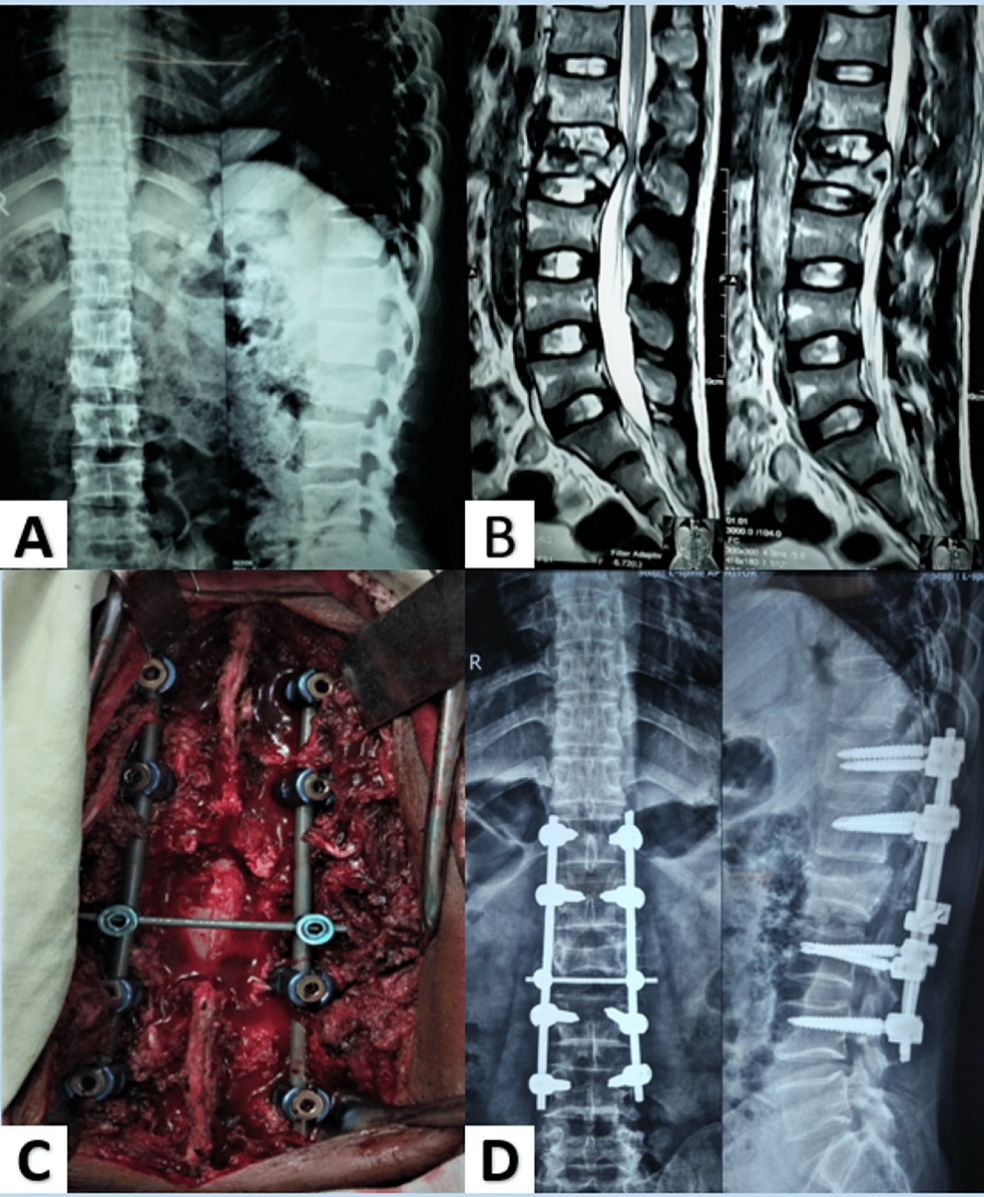
Long-segment posterior fixation. A. Preoperative X-ray of the lumbosacral spine anteroposterior and lateral view. B. T2-weighted sagittal section (MRI of the lumbosacral spine) showing burst # L2. C. Peroperative image showing long-segment pedicle fixation with cross-link. D. X-ray of the lumbosacral spine anteroposterior and lateral view at the final follow-up.

Figure 2 depicts a burst # L2 in a 28-year-old individual corrected with LSPF.

Postoperatively, all patients were immobilized in a thoraco-lumbosacral orthosis for six weeks. At one, three, six, and 12 months following surgery, as well as annually thereafter, all patients were examined. Radiological parameters and functional outcomes were evaluated by a third-party observer during each visit. All radiographic measurements, ASIA grading, Oswestry Disability Index (ODI), and Visual Analog Scale (VAS) were recorded preoperatively, postoperatively, and at every follow-up. The kyphotic angle, kyphotic deformation, and Beck index were used to assess the radiographic outcomes. The angle between the upper and lower intact vertebral endplates was used to calculate the kyphotic angle. The angle between the inferior and superior endplate of the fractured vertebrae was used to calculate kyphotic deformation. The Beck index is the difference between the anterior and posterior vertebral heights [16]. A 100-point VAS scale was used to assess pain levels. The ODI was used to assess the functional outcomes. ASIA grading was used to assess neurological recovery.

Statistical analysis

SPSS version 25.0 (IBM Corp., Armonk, NY, USA) was used for statistical analysis. The normal variable distribution was determined using the Kolmogorov-Smirnov test. The independent-sample t-test was used to compare the mean data. For each group, an analysis of variance (ANOVA) design was used to compare different time readings of radiographic parameters. Non-parametric statistical methods were used to analyze heterogeneous variables. The non-parametric variables were analyzed using the Mann-Whitney U test. The chi-square test was used to analyze categorical variables between groups. The statistical significance level was set at 0.05.

## Results

Table 1 illustrates the demographic details of patients. Regarding age and gender, there was no significant variation between the two groups. In addition, no significant statistical difference was observed among occupation, mode of injury, and skeletal level of injury. The age range in both groups was 15-60 years. Overall, 23 (57.5%) patients belonged to the 15-30-year range in SSFIFL, and 20 (50%) patients in the LSPF group belonged to the 31-45-year range.

**Table 1:** Patient demographic data. SSFIFL = short-segment fixation including fracture level; LSPF = long-segment posterior fixation

	SSFIFL	LSPF	P-value
Number of patients	40	40	
Age in years	32.30 ± 11.85	33.13 ± 10.29	0.512
Sex (Male/Female)	32/8	35/5	0.363
Occupation	Day labor (45%/42.5%)		0.623
Mode of injury	Fall from a height (85%/80%)		0.183
Skeletal level of injury	L1 (52.5%/57.5%)		0.059

L1 was by far the most commonly involved skeletal level, followed by L2 with 21 (52.5%) cases and 13 (32.5%) cases in the SSFIFL group, respectively. L1 was also the most common level involved in the LSPF group with 23 cases (57.5%), followed by D12 with 10 (25%) cases.

According to the load-sharing classification, the mean score was 4.68 ± 0.888 and 4.75 ± 0.776 for the SSFIFL and LSPF groups, respectively (p = 0.689). Fractured vertebrae were found unstable according to Punjabi and White [27] scores of 5.55 ± 0.677 and 5.70 ± 0.687 for the SSFIFL and LSPF groups, respectively (p = 0.328). The mean follow-up period was 28.48 ± 3.735 months for the SSFIFL group and 29.50 ± 3.113 months for the LSPF group (p = 0.186).

Table 2 provides a quick overview of radiological information preoperatively, postoperatively, and at the most recent follow-up for groups I (SSFIFL) and II (LSPF). Final radiographic data for both groups showed no statistical significance (p > 0.05), although the initial postoperative kyphotic angle and beck index was statistically significant.

**Table 2:** Radiological parameters and their significance. SSFIFL = short-segment fixation including fracture level; LSPF = long-segment posterior fixation

Index	SSFIFL	LSPF	P-value
Preoperative kyphotic angle	22.75 ± 4.53	21.63 ± 8.31	0.380
Postoperative kyphotic angle	3.98 ± 1.44	5.58 ± 2.07	0.000
Final kyphotic angle	9.13 ± 3.04	10.18 ± 3.35	0.120
Preoperative kyphotic deformation	22.75 ± 4.69	21.48 ± 5.89	0.351
Postoperative kyphotic deformation	9.00 ± 2.90	8.48 ± 2.35	0.550
Final kyphotic deformation	10.53 ± 3.11	9.88 ± 2.22	0.360
Preoperative Beck index	0.56 ± 0.12	0.59 ± 0.146	0.391
Postoperative Beck index	0.80 ± 0.08	0.85 ± 0.087	0.001
Final Beck index	0.769 ± 0.08	0.77 ± 0.095	0.776

At the last follow-up, the two kinds of stabilization were matched with the kyphotic angle, kyphotic deformation, and Beck index for every point of time using a one-way ANOVA model that was not statistically significant with p-values of 0.146, 0.286, and 0.870, respectively.

In both groups, preoperatively, most cases were graded as ASIA-C and finally ASIA-E with no statistical significance (p = 0.116 and 0.329, respectively). ASIA grade improvement was approximately the same in both stabilization procedures. One grade improvement was observed in 26 (65%) cases in the SSFIFL group and 23 (57.5%) cases in the LSPF group. Two grade improvements were observed in 13 (32.5%) cases in the SSFIFL group and 16 (40%) cases in the LSPF group (p = 0.781). No neurological deterioration was observed after surgery. However, one case in group II showed no neurological improvement at the final follow-up.

Regarding functional improvement, the ODI and VAS were improved significantly from the preoperative to postoperative state in both stabilization procedures. However, the independent-sample t-test showed no statistical significance between means at the final follow-up (p = 0.567 and 0.161, respectively). The chi-square test conducted for the categorical improvement of ODI was also not significant (p = 0.238).

The average operative time was 119.58 ± 19.93 and 131.35 ± 18.85 minutes in the SSFIFL and LSPF groups, respectively (p = 0.008). The mean blood loss was 350.38 ± 31.26 mL in the SSFIFL group and 427.31 ± 49.62 mL in the LSPF group (p = 0.000). Hence, blood loss and operative time were significantly higher in the LSPF group.

The rate of complication was statistically insignificant between the two stabilization groups (Pearson chi-square test, p = 0.416). The main complication in both stabilization procedures was a superficial infection which was resolved spontaneously. Screw breakage was found in two (5%) cases in the LSPF group at the final follow-up and subsequently removed.

Finally, the mean cost of the implant in USD was 258.38 ± 17.59 in the SSFIFL group and 406.05 ± 24.31 in the LSPF group which was statistically significant (p < 0.05).

## Discussion

Regarding thoracolumbar burst fractures, there are currently no scientific treatment regulations dictating the appropriate surgical approach and instrumentation technique [14,23,27-29]. Because it offers a stiffer fixation with reduced morbidity, posterior pedicle fixation is the most commonly used type of surgery for thoracolumbar fractures [17]. However, the choice between short- and long-segment pedicle screws remains unclear in this scenario [17,23,28-31]. Conventional LSPF provides adequate stabilization to allow patients to be mobilized earlier than usual and return to normal activities. It also lowers the risk of recurrent kyphosis, implant breakdown, and delayed neurological deficit [23]. On the other hand, a motionless spine can result from at least five levels of fixation. SSPF has recently gained popularity due to a theoretical reduction in surgery time and implant expenses and the potential to save more motion segments [23,30,31]. According to some studies, SSPF is the best option for flatback disorder and loss of lumbar lordosis [32]. In addition, the main drawbacks of the procedure were insufficient lengthy reduction and instrumentation failure with postoperative kyphosis [33]. Some surgeons recommend inserting pedicle screws at the fractured level to strengthen the instrumentation [23,30] to avoid potential complications with both LSPF and SSPF.

According to a recent meta-analysis [28], one procedure had no substantial advantage over the other (SSPF versus LSPF). Tezeren et al. discovered that long-segment instrumentation produced better results in terms of local kyphosis and anterior body height [33]. However, there was no significant statistical disparity in clinical outcomes.

Several studies have concluded that fracture-level screw integration produces superior results (statistically meaningful) compared to the bridging group in terms of the correction of the local kyphosis angle and anterior body height [23,29].

With and without the use of fracture-level screws, several recent retrospective studies have been conducted [30,31] and reported better correction by incorporating screws in fractured vertebrae in comparison to no screws.

Some studies have suggested a combined SSPF and staged anterior column reconstruction to achieve complete initial restoration of the weight-bearing column and correction of kyphosis [17]. However, it necessitated two surgeries, which added to the expense and higher perioperative morbidity and pain. Hence, they are not widely accepted in neurologically intact patients [34]. The study also concluded that this combined approach is indicated where the posterior approach by itself is inadequate to withstand a biological axial force; hence, effective restoration of the anterior weight-bearing block was required to avoid a secondary decrease in correction and implant failure.

Short-segment posterior fixation (SSFIFL) incorporating the fracture level was compared with long-segment fixation (LSPF) in this prospective study. We found no significant differences in demographics across groups in terms of age, gender, trauma etiology, fracture level and type, neurologic status, and follow-up time. At the preoperative evaluation, homogeneity was also observed in the kyphotic angle and Beck index.

Spinal injuries are much more common among young people than in any other age group. In this study, the mean age was 32.30 ± 11.85 in the SSFIFL group (range = 15-30 years) and 33.13 ± 10.29 years in the LSPF group (range = 31-45 years) with no significant difference (p = 0.512). The mean age was 30.74 ± 10.31 in the study by Raja (2010) [35,36].

Of the 80 cases, the majority of the patients were males (32 (80.00%) in the SSFIFL group and 35 (87.5%) in the LSPF group). There was no significant difference between these groups (p = 0.363). According to previous studies, men are more susceptible to trauma than women [22], which was also cited in a series of 50 patients, with 86% male patients [36].

In this study, the most frequent reason for injury was a fall from a height which accounted for 85% of cases. In the studies by Raja and Lee et al. 92% and 59.25% of cases, respectively, were attributed to a fall from a height [36,37]. Another study, however, found that the most frequent reason for injury was road traffic collisions [14].

The most commonly injured level was L1 (52.50% (21) in the SSFIFL group, and 57.5% (23) in the LSPF group). The literature reinforces this finding [14].

Using screws inserted at the fracture level, as hypothesized by Guven et al. [23], this research proves that it is possible to perform intraoperative fracture reduction and correction of sagittal deformity with relative ease. Moreover, it can prevent loss correction over time [30,31].

The mean kyphotic angle in SSFIFL and LSPF groups at the last follow-up was 9.13 ± 3.04° and 10.18 ± 3.35° (p = 0.120) with loss of correction of 5.15 ± 2.54° and 4.65 ± 2.42° (p = 0.371), respectively, which was insignificant in terms of kyphotic angle correction. Similar results were reported in a comparative study [31], where the preoperative mean kyphotic angle was 20.96° ± 4.74° in SSFIFL and 22.59° ± 5.890 in LSPF (p = 0.234). The mean kyphotic angle at the last follow-up was 15.97 ± 5.62° in SSFIFL and 17.76 ± 11.22° LSPF groups, which was also not statically significant (p = 0.579).

Regarding the radiological markers evaluated (Cobb angle and kyphotic deformation), the LSPF group outperformed the SSPF group [12]. Short-segment stabilization with extra screws at the fracture level, on the other hand, promoted kyphosis adjustment, sagittal index, and a compression ratio of the anterior vertebral height [31] in thoracolumbar burst fractures.

The mean preoperative kyphotic deformity was 22.75 ± 4.690 in the SSFIFL group and 21.48 ± 5.89° in the LSPF group (p = 0.351); the immediate postoperative kyphotic deformity was 9° ± 2.9° and 8.48° ± 2.35°, respectively (p = 0.550); and the final follow-up kyphotic deformity was 10.53 ± 3.11° and 9.88 ± 2.22°, respectively, which was insignificant (p = 0.360). In the SSFIFL group, the average preoperative wedge angle was 23.0° ± 8.1°. Corrections were made to 9.7° ± 6.20 (p=0.000), and kyphosis (mean = 1.2°) [30] was lost which coincides with the present study.

The mean preoperative Beck index was also insignificant between both groups during the preoperative (p = 0.391) period, postoperative period, and final follow-up (p = 0.776). Sapkas et al. [12] reported similar results with no statistical significance.

Regarding functional improvement, the ODI improved from a preoperative score of 67.20 to a final of 25.08 in the SSFIFL group and from a preoperative score of 68.35 to a final of 23.85 in the LSPF group (p = 0.587). According to a recent meta-analysis [34], five studies evaluated ODI and revealed no significant difference (p = 0.36). VAS status for pain also improved from preoperative to the final follow-up but without statistical significance (p = 0.161).

Regarding complications, significant variation was not found between the two groups (p = 0.416). The main complication in both stabilization procedures was a superficial infection which resolved spontaneously. Screw breakage was found in two (5%) cases in the LSPF procedure at the final follow-up and was subsequently removed. A meta-analysis of seven studies revealed no statistical significance in terms of total complication rate (p = 0.31) [33].

Another recent meta-analysis [38] revealed significantly reduced operative time in the SSPF group than in the LSPF group (p = 0.006). The average operative time in our study was 119.58 ± 19.93 and 131.35 ± 18.85 minutes in the SSFIFL and LSPF groups, respectively (p = 0.008), which is similar to the previously reported meta-analysis.

The mean blood loss was 350.38 ± 31.26 mL in the SSFIFL group and 427.31 ± 49.62 mL in the LSPF group (p = 0.000). Hence, blood loss was significantly more in LSPF groups. However, three other trials reported statistically insignificant blood loss (p = 0.23) with high heterogeneity (I^2^ = 79%; p = 0.0080) [36].

In this study, patients’ neurological status improved significantly at the final follow-up in both stabilization procedures but the improvement was not statistically significant (p = 0.781) between the procedures. Similar results (p = 0.579) were reported in a comparative study [39]. This study showed no decrease in the ASIA impairment scale suggesting the credibility of the procedure.

Finally, the mean cost of the implant in USD was 258.38 ± 17.59 in the SSFIFL group and 406.05 ± 24.31 in the LSPF group which was statistically significant (p < 0.05). According to our study, the LSPF group had to spend approximately USD138 more than the SSPF group.

Short instrumentation with fracture level inclusion has almost the same correction loss as a long construct in our observation. For example, in this series, SSFIFL had several advantages, such as a shorter operative time and the ability to maintain a correct sagittal alignment. In addition, both groups had similar neurological outcomes.

The study’s single-center design, limited sample size, and shorter follow-up period are all potential limitations. ­Therefore, more high-quality, multicenter, randomized controlled trials with longer follow-ups need to be conducted to select the appropriate surgical modality in patients with thoracolumbar burst fractures. Moreover, bone mineral density analysis was not routinely performed to exclude the grade of osteoporosis between the two groups which might have influenced the stability as well as loss of correction to a great extent.

## Conclusions

There was no substantial difference between the SSFIFL and the LSPF regarding clinical or radiological outcomes. Therefore, picking one over the other is difficult. The decision must be made with care and according to the unique circumstances of each patient. Two or more vertebral segments can be saved by incorporating a fracture level into a short-segment fixation technique (SSFIFL). Furthermore, the SSFIFL technique has a lower operative time, blood loss, and implant cost than the LSPF technique, making it a better surgical option.
